# Language-Based Detection of Depression with Machine Learning: Systematic Review and Meta- Analysis

**DOI:** 10.21203/rs.3.rs-8067325/v1

**Published:** 2025-11-18

**Authors:** Hadar Fisher, Nigel M. Jaffe, Kristina Pidvirny, Anna O. Tierney, Mia S. Vaidean, Poorvesh Dongre, Christian A. Webb

**Affiliations:** McLean Hospital; McLean Hospital; McLean Hospital; McLean Hospital; McLean Hospital; Virginia Tech; McLean Hospital

**Keywords:** Depression, Artificial intelligence, Natural Language Processing, Machine learning

## Abstract

Early detection of depression is critical for timely intervention. Natural language processing (NLP) and machine learning (ML) approaches have increasingly been used to automatically detect depression from text data, yet comprehensive evidence regarding their diagnostic performance remains limited. We systematically reviewed and meta-analyzed studies applying NLP and ML to identify depression from spoken or written language. Six electronic databases and additional sources were searched, yielding 892 full-text articles, of which 123 met inclusion criteria. One representative result per dataset was selected for quantitative synthesis, resulting in 50 independent studies. Pooled accuracy across studies (k = 43; n = 40,983) was 0.80 (95% CI, 0.76–0.83). Precision (k = 28) was 0.78 (95% CI, 0.72–0.83), recall (k = 33) 0.76 (95% CI, 0.68–0.83), AUC (k = 14) 0.79 (95% CI, 0.70–0.85), and balanced accuracy (k = 16) 0.71 (95% CI, 0.63–0.78). Subgroup analyses showed significant differences by language, text source, feature type, and classifier (all p < .001). Accuracy was highest in studies using structured clinical interviews, non-English languages, and linguistic or embedding-based features. However, in one-at-a-time meta-regressions, only text source remained a significant predictor (QM(3) = 8.78, p = .032), explaining 13.6% of the between-study variance. Publication bias was minimal. Automated depression detection from text shows promising performance with substantial heterogeneity. Performance varies by language, data source, feature extraction, and model type. Findings highlight both current limitations and potential of text-based depression detection and underscore the need for methodological standardization and validation before clinical use.

## Introduction

Depression is among the most prevalent psychiatric disorders worldwide and a leading cause of disability.^1^ Its global prevalence has risen steadily over the past three decades, with the United States alone seeing depressive symptoms increase approximately threefold from 2017 to 2020.^2^ Despite this high burden, depression remains substantially underdiagnosed and undertreated, with many individuals never seeking professional help or doing so only after symptoms have become severe and impairing.^3^ Untreated depression is associated with worse prognosis, highlighting the importance of early identification for timely intervention and improved outcomes.^4,5^ Emerging digital health tools offer opportunities to expand access and may enable earlier depression detection.^6^

One promising avenue for automated depression detection lies in the analysis of language.^7^ Advances in natural language processing (NLP) and machine learning (ML) now enable scalable, automated analysis of linguistic data to detect depression.^8^ The use of NLP and ML to detect depression has grown exponentially in recent years. However, despite this progress, it remains uncertain how accurate these approaches are overall and whether their performance generalizes reliably across different contexts. Importantly, much of this work has been published in computer science journals, making it less accessible to psychiatric researchers and clinicians who might benefit from these insights. Clinical researchers must be engaged and informed to critically evaluate, guide, and translate these tools into meaningful clinical use.

Several prior reviews have mapped the landscape of NLP and ML approaches for detecting depression, summarizing common text sources, feature extraction strategies, and model architectures.^8–15^ However, despite the rapid expansion of this research area, none of these reviews quantitatively evaluated the accuracy of these models across studies. In this systematic review and meta-analysis, we evaluated the performance of NLP- and ML-based models in detecting depression using datasets where depression status was determined independently (e.g., through validated questionnaires or clinical diagnoses). We further examined study-level moderators, including language, text source, feature type, and model class.

## Methods

This systematic review and meta-analysis evaluated the performance of NLP and ML models for detecting depression from text data. The review followed the Preferred Reporting Items for Systematic Reviews and Meta-Analyses (PRISMA) reporting guidelines (**Table S1**).^16^ The study protocol was preregistered with the International Prospective Register of Systematic Reviews (PROSPERO; ID CRD42024513390).

Screening and review management were conducted using Covidence software (Veritas Health Innovation, Melbourne, Australia). In the first and second phases (title and abstract review, full text review), two independent raters screened each record. Disagreements were resolved by consensus between reviewers. To ensure consistency and reliability, all reviewers participated in six structured training sessions prior to data collection: two sessions each for title screening, abstract screening, and data extraction. Meetings continued periodically throughout the screening and extraction period.

### Data Sources and Search Strategy

We systematically searched both computer science and psychiatric databases to capture the multidisciplinary literature on this topic. Searches were conducted during March 2024 in ScienceDirect, IEEE Xplore, ACM Digital Library, Scopus, MEDLINE, PubMed, and PsycINFO. The search was supplemented in September 2025 by a hand search of reference lists and relevant reviews. The search string combined disorder-specific and computational terms: “depress*” AND (“language” OR “text”). Searches were restricted to peer-reviewed articles published in English.

### Study Eligibility Criteria

Inclusion criteria required that studies: (1) used adult participants’ (≥ 18 years) own spoken or written text data to detect (concurrent) depression; (2) employed formal measures of depression (e.g., structured clinical interview, self-report questionnaire, clinical diagnoses); (3) applied machine learning (ML) models to predict the person’s depression status (i.e., binary classification) or depression severity; (4) relied solely on textual input for model development and prediction (i.e., studies incorporating additional modalities such as audio, video, or demographic variables were excluded if they lacked a text-specific model); (5) were peer-reviewed and fully available in scientific databases; and (6) provided sufficient information to extract classification performance (e.g., accuracy, precision, recall, F1, or equivalent).

For the purpose of this review, we included only studies that evaluated model performance on an external sample (e.g., through cross-validation or independent test data), ensuring that reported estimates reflected true predictive capacity rather than overfitting to training data. Large Language Models (LLMs) that were used directly for text classification were not required to include additional validation, as these models are pretrained on large, diverse corpora and apply learned representations without task-specific parameter fitting, reducing the risk of overfitting. However, when LLMs were used solely for feature extraction (e.g., deriving sentiment or emotion scores) and the extracted features were subsequently entered into a separate classification or prediction model to detect depression, such studies were required to include a validation process to ensure the robustness and generalizability of the results.

Studies relying exclusively on social media text were excluded. We made the decision to exclude social media text in order to ensure the inclusion of high-quality studies with clear clinical relevance. Social media data pose several limitations^15^: first, depressed individuals may change their online activity, leading to underrepresentation and biased samples. Second, in many social media datasets, depression status is inferred directly from the text, for example, by external annotators rating whether the text contains depression-related content, so the same material is used both to define and to predict depression. This circular annotation strategy undermines label validity and limits generalizability to clinically assessed depression.

### Data Extraction and Risk of Bias Assessment

HF and two reviewers per study independently extracted data from eligible studies using a standardized template and assessed risk of bias using the ML-specific quality assessment tool developed by Ciharova et al.,^17^ adapted from established instruments (PROBAST,^18^ Cochrane RoB 2.0,^19^ ROBINS-I^20^). When studies reported results from multiple ML models (e.g., based on different feature sets or classifiers), we extracted the performance metrics of the model that achieved the highest performance. Any discrepancies were resolved in group discussion until consensus was reached. In addition to reading the full text of each article, extracted responses were validated using NotebookLM, which supported but never replaced reviewer judgment. This process was implemented to maximize accuracy and agreement while ensuring that decisions were grounded in direct review of the original studies.

Extracted variables included: study sample size; text source and language; depression measure; feature extraction method (e.g., statistical, linguistic, embeddings, transformer, hybrid); classifier type (traditional machine learning, neural, transformer); type of validation (e.g., k-fold cross validation, train-test split); and reported outcomes (e.g., accuracy, precision, recall, and other model performance metrics).

Given the absence of a standardized framework for evaluating the quality of machine learning studies, we adopted the quality assessment approach used in prior research on ML-based prediction models.^17^ In line with that framework, study quality was assessed across four domains: (1) adequate sample size (≥ 100 participants), (2) balanced class distribution in cases where participants are divided into multiple groups, such as depressed vs. non-depressed (no group more than ten times smaller than others), (3) appropriate model validation (model parameters tuned on a training set and evaluated on an independent test set), (4) use of a validated outcome measure assessing depression presence or severity (a.k.a., ground truth). These criteria were designed to minimize risk of overfitting and enhance generalizability to unseen data. Since using validation was an inclusion criterion, item 3 was not relevant and is excluded from the quality assessment table. See **Table S2** for the risk of bias table.

### Outcomes

The primary outcome was pooled classification accuracy, which quantifies the overall proportion of classifications made by the model that were correct, or in this case, the overall rate at which the model correctly identifies both depressed and non-depressed individuals. Secondary outcomes included precision (positive predictive value), sensitivity (recall), and the area under the receiver operating characteristic curve (AUC). Precision is the proportion of correctly predicted positive cases among all predicted positive cases, whereas sensitivity is the proportion of actual positive cases correctly identified. High precision reflects fewer false alarms, while high sensitivity reflects fewer missed cases. AUC considers both sensitivity and specificity in its calculation, showing the balance between predicting a positive outcome when the outcome is indeed positive and predicting a negative outcome when the outcome is indeed negative. Mathematically, it represents the area under the curve plotting sensitivity (true positive rate) against 1 – specificity (false positive rate). When possible, we also calculated balanced accuracy, defined as the average of sensitivity and specificity. By giving equal weight to correctly identifying positive and negative cases regardless of their prevalence, it provides a less sample-biased estimate of model performance, particularly in datasets where one class (e.g., depressed or non-depressed) is more frequent than the other. The F1-score was used for interpretation in the systematic review but was not included as an outcome in the meta-analysis. This is because it is a harmonic mean of two ratios that already contain partial information about sample size, making its sampling variance difficult to estimate for meta-analytic weighting.

### Statistical Analysis

We extracted both qualitative and quantitative data from each selected study. Meta-analysis was conducted for studies where data availability permitted summary estimation with 95% confidence intervals (CI). Accuracy and related performance metrics were first logit-transformed to stabilize variances and normalize distributions before pooling. Primary pooled estimates were obtained using random-effects models fit with maximum likelihood estimation. To assess heterogeneity, we used τ ^2^, which quantifies the absolute magnitude of the true between-study variance, and *I*^2^, which indicates the percentage of the total observed variance that is attributable to real differences in effect sizes rather than to sampling error. Publication bias was evaluated through visual inspection of funnel plots and the Galbraith (radial) plot, Egger’s regression tests, and the trim-and-fill method. As a sensitivity analysis, leave-one-out analyses assessed the influence of individual studies. We repeated the same procedure with precision, sensitivity and AUC.

To enable pooled estimation across studies that reported performance metrics but not underlying classification counts (i.e., confusion-matrix data), we reconstructed approximate event counts for AUC, precision, and recall following the same logic applied to accuracy. For each study, the number of “events” was computed as the reported metric multiplied by the total sample size, rounded to the nearest integer, with the remainder treated as “failures.” This reconstruction allowed standard error estimation based on the binomial distribution and ensured consistent weighting across studies with differing sample sizes. While AUC reflects overall discriminative ability and precision and recall represent class-specific performance, all three were analyzed using the same framework to ensure comparability. Using total sample size instead of class- or threshold-specific denominators provided a conservative approximation for variance estimation across studies. Effect sizes were calculated using logit transformation of proportions (PLO). A continuity correction of 0.5 was applied to stabilize estimates when proportions were 0 or 1. All pooled estimates and CIs were back-transformed to the probability scale for interpretability.

Subgroup analyses compared pooled accuracies across *language* (English, Chinese, Other), *source of text* (clinical interview, open-ended questions, communication, interaction with therapist), *feature extraction method* (simple, linguistic, embeddings, transformer, hybrid), and *classifier type* (traditional, neural, transformer). Between-group heterogeneity (Q_between_) was estimated using random-effects models with restricted maximum likelihood estimation. To examine whether these factors explained variability in accuracy, we then ran one-at-a-time meta-regressions with omnibus tests (QM) and R^2^ based on reductions in τ^2^. All analyses were performed in *R*, version 4.3.3, using the *metafor* package (version 4.8.0). Code is available at https://osf.io/x7tm9.

## Results

**Figure 1:**
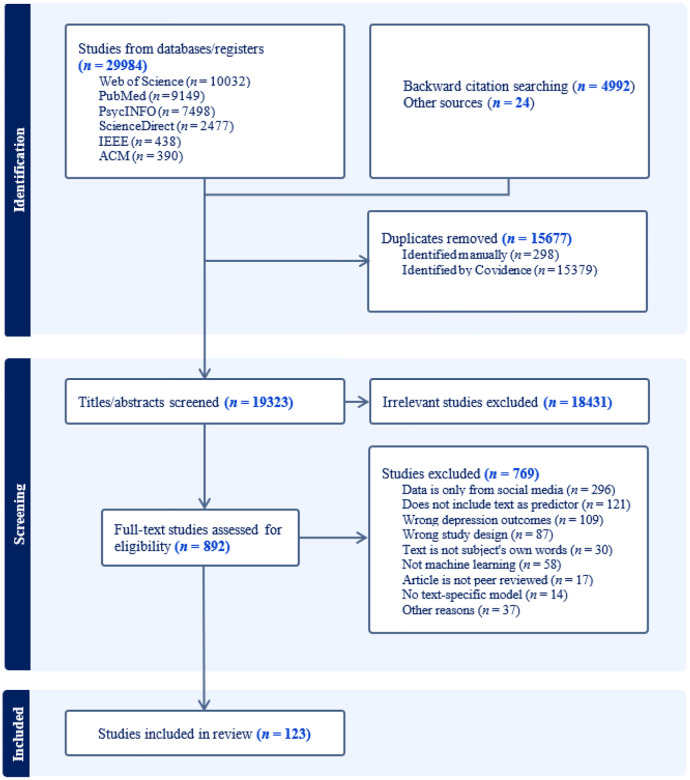
Flow Diagram of Study Selection

The search yielded 35,000 records. After removing duplicates and screening titles and abstracts, 892 full-text articles were reviewed, and 123 publications contributing 129 effects were included in the qualitative synthesis (Fig. 1). When articles reported results based on multiple datasets, each dataset-specific effect was treated as a separate study.

Over half of the included studies (73/129, 56.6%) used the Distress Analysis Interview Corpus (DAIC) or related datasets derived from it, including three studies that used DAIC alongside another dataset. The DAIC is a well-known benchmark dataset comprising semi-structured clinical interviews. Its Wizard of Oz (DAIC-WOZ) subset,^21^ released as part of the AVEC 2016 challenge, includes 189 interviews conducted by a virtual agent, with audio, video, and transcript recordings available. An extended version (E-DAIC)^22^ was later released for AVEC 2019, expanding the sample to 275 interviewed participants. In both datasets, depression severity was labeled using the Patient Health Questionnaire-8 (PHQ-8)^23^. Table 1 summarizes studies using unique datasets, and Table 2 summarizes those using DAIC data.

Across studies, a total of 35,171 unique participants (removing duplicates from overlapping datasets) contributed 58,413 text samples (e.g., utterances, transcripts, or messages). Publication years ranged from 2013 to 2025, with 95 articles (77.2%) published since 2020, reflecting growing interest in automated text-based depression detection.

### Dataset Characteristics^1^

Among the 56 studies using unique datasets, 20 (35.7%) analyzed English text, 14 (25%) analyzed Chinese text, and 22 (37.5%) examined other languages (e.g., Italian, Spanish, Turkish, Korean, Russian, German, Malay, Thai). One study (1.7%) combined Chinese and English datasets by translating the Chinese texts into English. The DAIC datasets included English-language clinical interviews.

The number of text observations ranged from 53 to 15,950 (mean = 1,216; median = 210). Key categories of data sources included: Structured clinical interview (19/56, 33.9%), responses to open-ended questions (e.g., “ Describe your weekend activities”; 27/56, 48.2%), text messaging and chat logs (6/56, 10.7%) or interaction with therapists (i.e., text-based therapy; 4/56, 7.1%).

### Outcome Formulations

The great majority of studies framed automated depression detection as a binary classification task (100/129, 77.5%). In these cases, models were trained to identify whether an individual (or a given text sample) was produced by a depressed vs. non-depressed person. Typically, “depressed” status was defined using a clinical cutoff on a depression severity questionnaire (for example, a PHQ-9 score ≥ 10) or based on a formal diagnostic evaluation.

### NLP feature extraction and classification methods^2^

To train ML models to predict depression, text should be converted into numeric values that will be used as the input. Broadly, feature extraction methods fell into four categories: (1) Simple textual features (12/129, 9.3%): Unstructured representations such as Bag-of-Words (BoW) or Term Frequency-Inverse Document Frequency (TF-IDF) term vectors, which convert text into numerical frequency-based vectors without incorporating external knowledge. (2) Lexicon-based linguistic features (16/129, 12.4%): Specific variables derived from psychological dictionaries or lexica (e.g., LIWC, EmoLex, SenticNet). These typically involve counting the occurrences of words in predefined semantic, emotional, or psychological categories (e.g., number of sad words in the text). (3) Pre-trained word embeddings (31/129, 24.0%): Dense, distributed vector representations of words or documents learned from large text corpora, excluding transformer-based models. Common examples included Word2Vec and GloVe embeddings, which represent words based on their co-occurrence patterns in large datasets. (4) Transformer-based language model features (48/129, 37.2%): Deep text representations from large pre-trained models (e.g., BERT, GPT) that use attention mechanisms to capture relationships between words across entire sentences, enabling a more nuanced understanding of context and meaning. Studies also employed hybrid feature approaches, combining multiple feature types to enrich the model’s input (22/129, 17.0%).

In line with broader NLP trends, classification methods evolved from traditional machine-learning models to deep learning approaches capable of directly interpreting and learning from raw text. About one-third of studies (44/129, 34.1%) used traditional classifiers such as support vector machines, logistic regression, naïve Bayes, or random forests. A large portion (56/129, 43.4%) of the studies employed recurrent neural network architectures, most commonly Long-Short-Term Memory (LSTM) or bidirectional LSTM, often augmented with attention mechanisms to capture the temporal dependencies among words. By the early 2020s, transformer-based models (29/129, 22.4%) became increasingly prevalent, with studies either fine-tuning pre-trained architectures (e.g., BERT, RoBERTa) for depression detection or using their contextual embeddings as features to separate classifiers. For example, Flores et al.^24^ found that while BERT embeddings alone achieved relatively modest performance (maximum F1: 0.58), combining them with an LSTM substantially improved results (average F1: 0.72).

### Meta-analysis results

**Figure 2:**
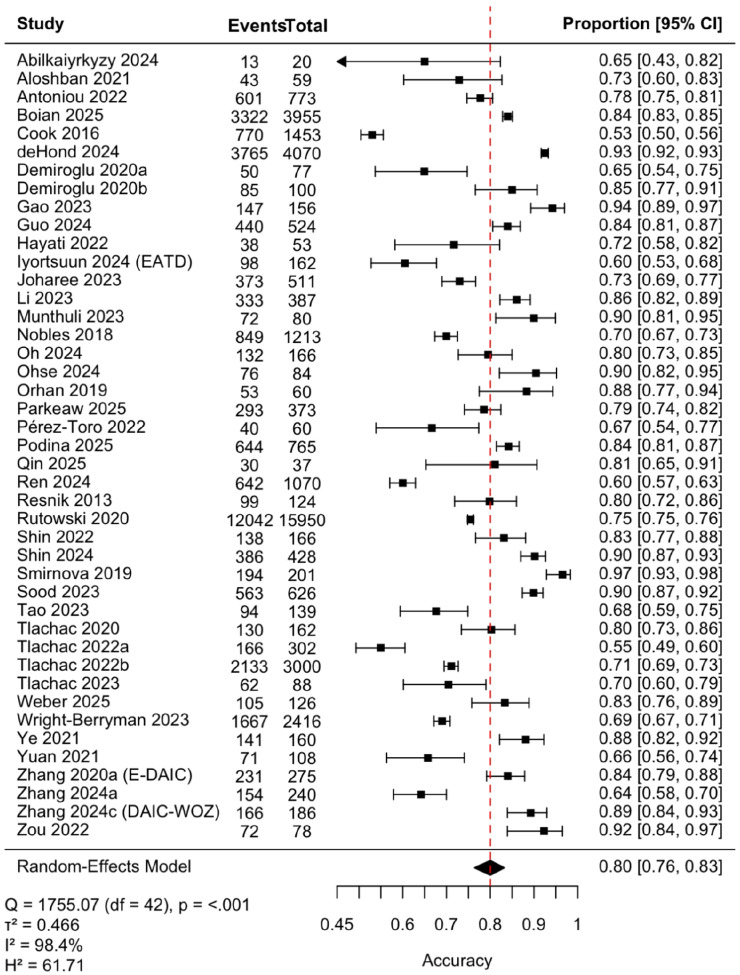
Forest plot of study-level classification accuracy with pooled random-effects estimate

**Figure 3:**
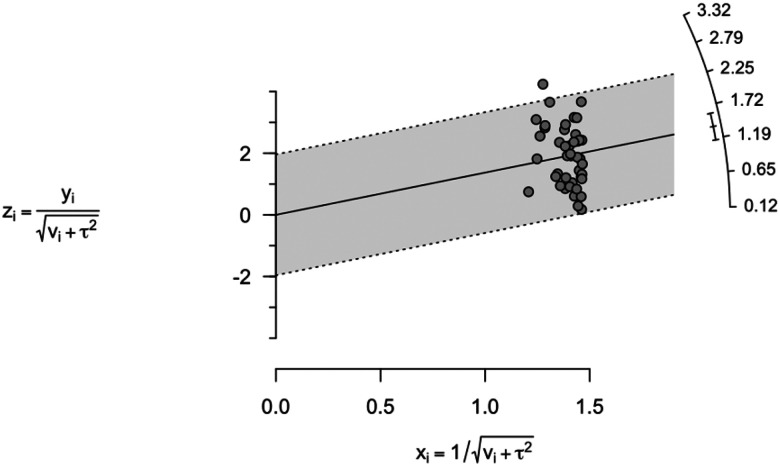
Galbraith (radial) plot assessing heterogeneity across studies in classification accuracy **Note.** The Galbraith (radial) plot displays each study’s standardized effect size (z_i_) against its **statistical precision** (1 / √(v_i_+ τ^2^)), where precision represents the inverse of the standard error, that is, the reliability of each study’s estimate. The x-axis indicates statistical precision, studies farther to the right are more precise (smaller SE), and the y-axis represents the standardized effect size, with points near the top or bottom deviating more from the pooled effect. The solid line represents the pooled effect from the random-effects model, and the shaded 95% confidence region indicates the expected range of variation. In this analysis, most studies fall within the shaded region, suggesting that the observed heterogeneity (I^2^ = 98.8%) reflects broad variability across studies rather than the influence of a single outlier.

**Figure 4:**
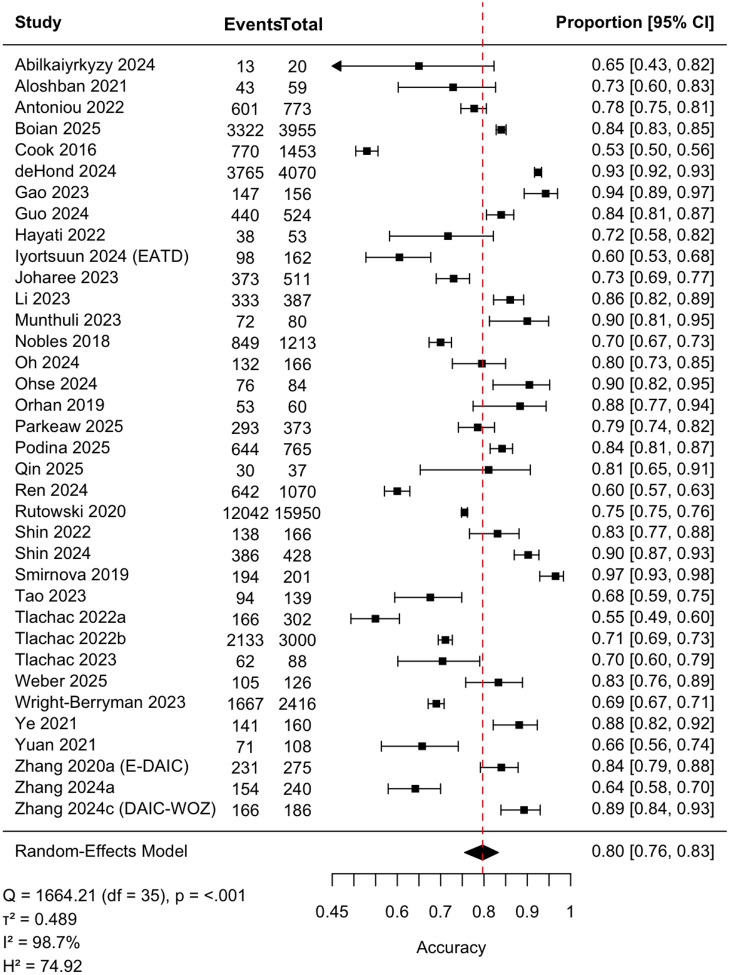
Forest Plot of Classification Accuracy from Sensitivity Analysis Excluding Studies

Forty-three studies (*n* = 40,983) were included in the pooled analysis of classification accuracy (Fig. 2). The pooled accuracy was 0.80 (95% CI, 0.76–0.83), with substantial heterogeneity (τ ^2^=.471, *I*^2^= 98.4%). Egger’s test was nonsignificant (*z* = 1.17, *p* = 0.25), and trim-and-fill identified no missing studies, indicating no evidence of publication bias. Leave-one-out analyses demonstrated stability (range: 0.79–0.80; **Table S3**), and the Galbraith plot (Fig. 3) revealed no extreme outliers, confirming that heterogeneity reflects broad between-study variability rather than undue influence from individual studies. As a sensitivity analysis, we also excluded seven studies in which accuracy was approximated from other reported metrics (*k* = 36), yielding an identical pooled estimate (0.80, 95% CI, 0.75–0.83) and similarly high heterogeneity (Fig. 4).

### Moderator analysis

There were significant between-group differences based on language (*Q*_*between*_= 153.24, *p* < .001). Pooled classification accuracy was highest for studies conducted in languages other than English or Chinese (*k* = 8, Accuracy = 0.82, 95% CI, 0.76–0.86), followed by those in Chinese (*k* = 9, Accuracy = 0.81, 95% CI, 0.70–0.88), and studies in English (*k* = 16, Accuracy = 0.77, 95% CI, 0.71–0.83). Between-group differences were also significant for text source (*Q*_*between*_=187.05, *p* < .001). Studies using structured clinical interviews (*k* = 18) demonstrated the highest pooled accuracy (0.84, 95% CI, 0.81–0.88), followed by communication-based interactions (*k* = 5, Accuracy = 0.79, 95% CI, 0.66–0.88), open-ended questions (*k* = 18, Accuracy = 0.75, 95% CI: 0.68–0.81), and finally therapist-patient interactions (*k* = 2, Accuracy = 0.70, 95% CI, 0.50–0.84). A significant between-group difference was observed based on feature type (*Q*_*between*_= 164.62, p < .001). Linguistic features produced the highest pooled accuracy (*k* = 5, Accuracy = 0.86, 95% CI 0.75–0.93), followed by embeddings-based features (*k* = 6, Accuracy = 0.84, 95% CI, 0.75–0.90), transformer (*k* = 18, Accuracy = 0.81, 95% CI, 0.75–0.85), simple features (*k* = 7, 0.75, 95% CI, 0.65–0.82), and hybrid features (*k* = 7, Accuracy = 0.74, 95% CI, 0.63–0.83). Classifier type also showed a significant between-group effect (*Q*_*between*_= 166.25, p < .001). Transformer-based and traditional classifiers performed similarly, both with Accuracy = 0.81 (transformers: *k* = 14, 95% CI, 0.74–0.87; traditional: *k* = 21, 95% CI, 0.76–0.85), outperforming neural networks (*k* = 8, 0.72, 95% CI, 0.63–0.80).

In one-at-a-time meta-regressions, only text source was significant (QM(3) = 8.78, *p* = .032), accounting for 13.6% of the variance. Other moderators including risk of bias, sample balance, language, feature type, classifier, and sample size (log N) did not significantly predict variability in accuracy (all p > 0.17; R^2^= 0–7.7%).

### Secondary analysis

#### Precision.

Twenty-eight studies (*n* = 31,644) yielded a pooled precision of 0.78 (95% CI, 0.72–0.83) with high heterogeneity (τ ^2^=.731,*I*2 = 99.1%, **Figure S1**). Leave-one-out analyses were stable (range = 0.77–0.79, **Table S4**).

#### Recall.

Thirty-three studies (*n* = 47,738) produced a pooled recall of 0.76 (95% CI, 0.68–0.83) with high heterogeneity (τ ^2^ =1.42 *I*^2^= 99.7%, **Figure S2**). Leave-one-out analyses confirmed stability (range = 0.75–0.77, **Table S5**).

#### AUC.

Fourteen studies (*n* = 39,412) showed a pooled AUC of 0.79 (95%CI: 0.70–0.85) with high heterogeneity (τ ^2^=0.66 *I*^2^= 99.6%, **Figure S3**). Leave-one-out analyses were stable (range = 0.76–0.81, **Table S6**). Across all three metrics, precision, recall, and AUC, Egger’s tests were nonsignificant, and trim-and-fill analyses indicated that no studies were missing, suggesting no evidence of publication bias.

#### Balanced accuracy.

Sixteen studies (*n* = 31,661) showed a pooled AUC of 0.71 (95%CI: 0.63–0.78) with high heterogeneity (τ ^2^ =0.54 *I*^2^= 99.4%, **Figure S4**). Leave-one-out analyses were stable (range = 0.70–0.74, **Table S7**). Egger’s test was nonsignificant, and trim-and-fill analyses suggested that only one study should be imputed, which slightly reduced the pooled balanced accuracy estimate from 0.71 to 0.70 (**Figure S5**).

## Discussion

This study revealed a substantial increase in studies using NLP- and ML-based approaches for automated depression detection. Among studies included in the quantitative synthesis, models achieved a pooled accuracy of 80%, correctly distinguishing depressed from non-depressed individuals in roughly four out of five cases. For comparison, meta-analyses using resting-state fMRI found similar accuracy (~ 80%)^25^, whereas those using wearable AI reported higher accuracy (~ 89%)^26^. Yet given the substantial heterogeneity, results should be interpreted cautiously.

Subgroup analyses showed significant differences in accuracy across several factors. Studies conducted in English yielded lower accuracy (77%) than those in Chinese or other languages, possibly reflecting linguistic or cultural differences^27^ or dataset-specific effects. This finding warrants further investigation into whether cultural and language factors affect automated detection. Models trained on structured clinical interviews achieved the highest accuracy (84%), whereas those analyzing free-form patient–therapist conversations performed worse (70%), suggesting that direct questioning about mood elicits clearer linguistic signals of depression.

Interestingly, lexicon-based features showed the highest pooled accuracy (86%), outperforming more complex or hybrid models (74%). One explanation is that targeted linguistic markers of depression, (e.g., frequent use of negative emotion words or first-person pronouns) are robust across contexts, allowing simpler models to perform well. However, this finding is based on few studies and requires further validation. Traditional machine learning models performed comparably to transformer-based models (81%), potentially indicating a performance ceiling, possibly due to limited input text that provides insufficient linguistic signal. Other factors may include between-person variability in how depression is expressed, small and imbalanced datasets, insufficient transformer fine-tuning, shallow linguistic cues in the data collection task, and differences in model optimization or evaluation procedures. These factors may constrain even advanced models, underscoring the need for richer longitudinal data and more personalized and context-aware modeling approaches. Consistent with this interpretation, only the type of text source significantly explained some between-study variance in meta-regression (accounting for 13.5% of heterogeneity), suggesting that depression-focused linguistic content may be more critical than model complexity for detection accuracy.

Secondary analysis reveals a pooled AUC of 0.79, suggesting that NLP models can detect depression from text at a level that could be clinically useful, especially for early screening or augmenting clinicians’ assessments. The pooled precision (0.78) and recall (0.76) suggest that, on average, models show a reasonable balance between correctly identifying depressed individuals and avoiding false positives. However, there was substantial variability across studies, likely because these metrics are complementary, and researchers can optimize one at the expense of the other. This trade-off means that while some models favor higher sensitivity to reduce missed cases, others prioritize precision to avoid false alarms.

This study is one of the few systematic reviews and the first meta-analyses to quantitatively evaluate NLP and ML techniques for detecting depression. Engaging clinical researchers in this rapidly evolving field is essential to ensure these tools are properly understood, evaluated, and directed toward real clinical needs. To maintain a high standard, we included only studies that validated their models against independent measures of depression. Nonetheless, several limitations should be acknowledged. The high heterogeneity means the pooled accuracy should be interpreted cautiously, as individual study results varied widely. Although moderator analyses explained some variability, much remains unexplained, likely reflecting differences in validation methods, preprocessing, or sample characteristics.

Furthermore, many papers did not report all the standard classification metrics. The field would benefit from more standardized reporting. Relatedly, accuracy alone can be misleading in imbalanced datasets where high accuracy may simply reflect the majority (non-depressed) class rather than true model performance.

An additional limitation is that over half of studies used variants of the DAIC corpus, meaning a large portion of the literature is built on the same or very similar data, which limits generalizability. While the DAIC is a valuable benchmark, there is a clear need for new datasets that cover different populations, languages, and modes of communication.

Gender differences were not examined, as few studies reported this information. Given that depression is more prevalent among women, they were likely overrepresented in training data, potentially biasing model performance and increasing misclassification risk for men.^28^ Finally, automated detection differs conceptually from tracking within-person change. Identifying who is depressed does not necessarily translate to monitoring fluctuations or improvement over time, which may rely on different linguistic dynamics.

In summary, NLP and ML can detect depression with good accuracy across a variety of settings. The growing interest since 2020 has yielded many promising approaches. Advancing the field will require greater standardization in reporting, the use of more diverse datasets, and explicit attention to gender and cultural fairness to address the substantial heterogeneity observed. With continued refinement and validation, text-based depression detection systems have the potential to complement traditional assessment methods and broaden access to mental health screening.

## Supplementary Material

This is a list of supplementary files associated with this preprint. Click to download.

- SupplementNJfinal.docx

## Figures and Tables

**Table 1:** Description of Studies Included in the Systematic Review and Meta-Analys

Study	Language	N	Diagnosis	Source of text	Features extraction method	Outcome	Type of classification model	Validation	Accuracy	F1	AUC	Specificity	Precision	Recall/Sensitivity
Abilkaiyrkyzy 2024^29^	English	20 (+ 275 trained on E-DAIC)	Mildly depressed: 8, Moderately depressed: 6 Not depressed: 4	Open-ended questions	Transformer (BERT tokenizer and LanguageModelFeaturizer)	PHQ-9	Fine-tuned BERT sequence classifier for multi-class depression severity (Softmax output).	Trained on E-DAIC tested on sample of 20 university students	0.65					
Aloshban 2021^30^	Italian	59	Depressed: 29 Not Depressed: 30	Interviewed about everyday life aspects (e.g., activities in the weekend of interaction with family members)	Embedding (Wikipedia2Vec)	Professional psychiatrists’ diagnosis	BiLSTM	5-fold cross validation	0.729	0.619			1	0.448
Antoniou 2022^31^	English	773 (270)	Depression/stress: 356 sessions of 184 patients. Other problems: 417 sessions of 86 patients	Interaction with therapist (text-based counseling)	LIWC	Patients report presenting problem before the first interaction	Quadratic discriminant analyses	5-fold cross validation	0.778	0.71	0.76			
Banerjee 2021^32^	English	1999	Unclear, 71.4% from the data before cleaning	Open-ended questions	Embedding (doc2vec); Affective features; Word polarity; linguistic tags (e.g., Proper Noun Tag, Singular Noun Tag)	PHQ-9	CNN-Dynamic Attention	60-20-20 random train-validation-test split		0.644				
Boian 2025^33^	Romanian	3955 (861)	Per-item classification Not at all (NO): 457 Several days (SD): 1,063, More than half the days (HA): 446, Nearly every day (EV): 442 or Irrelevant (IR): 227	Clinical interview (conducted by aiCARE chatbot)	TF-IDF	PHQ-9	Logistic regression	Split to train and test set Train: 1320 Test: 2635	0.840	0.80		0.80	0.80	0.80
Burkhardt 2022^34^	English	13327 (6551)	Unclear	Interaction with therapist	LIWC; Embedding (BERT-based model for GoEmotions was used to extract emotion features)	PHQ-9	Random forest	**Training set** (80%): 4,913 patients, 10,006 observations **Test set** (20%): 1,638 patients, 3,321 observations		0.520	0.67		0.612	0.453
Cao 2025^35^	Chinese	50 (the full sample included 100 patients the best model included 50)	Very severe 37 Mild 27 Severe 19 Normal 13 Moderate 13	Clinical interview	LLM (Qwen2.5-7B-Instruct)	HAMD-17	LLM: Qwen2.5-7B-Instruct fine-tuned with LoRA	leave-one-out cross-validation		0.61			0.61	0.61
Chen 2024^36^ (CMDC) (Same as Zou 2023)	Chinese	78	Depressed: 26 Not depressed: 52	Clinical interview	Transformer (the Chinese-BERT); Xmnlp: Word-level features: ratios of adjectives, adverbs, exclamations, verbs, auxiliary words, modal particles, and total word count; Sentence-level features: number of sentences, ratio of positive and negative sentences, and overall sentiment score; Lexical-emotion feature: proportion of modal words	MINI	IIFDD	5-fold cross-validation	0.87*	0.8			0.82	0.79
Chen 2024^36^ (EATD) (Same as Shen 2022)*	Chinese	162	Depressed: 30 Not depressed: 132	General interview	Same as Chen 2024a (above)	The Self-rating Depression Scale (SDS).	IIFDD	3-fold cross-validation		0.45			0.36	0.70
Cohen 2023^37^	English	73 (68)	Depressed: 15 Control: 58	Interaction with an online agent, Tina	TDF-IDF	PHQ-9	SVM	leave-one-subject-out			0.54			
Cook 2016^38^	Spanish	1458	Depressed: 662 Not depressed: 796	Free-text responses to the question: “how do you feel today?”	n-gram	GHQ-12	Logistic regression	50% split to train and test	0.53	0.42		0.79	0.64	0.31
deHond 2024^39^	English	4070	Depressed: 127 Not depressed: 3943	Patient (cancer)-generated emails to their health care teams	Transformer (BERT)	ICD-9 and ICD-10 codes obtained from electronic health record data	LASSO logistic regression	Train (67%): 2713 Test (33%): 1357	0.925	0.091	0.54	0.95	0.925	0.13
Demiroglu 2020^40^	Turkish	77 (70)	Depressed: 50 records. Not depressed: 27 records	Interview, with 3 types of questions: neutral, positive, and negative.	Average length of the utterances, subjects in negative, positive, and neutral answers separately. Three-dimensional feature. Rate of speech for negative, positive, and neutral answers. Sentiments of the question-answer pairs.	BDI	SVM	Leave-on-out	0.65*	0.68			0.76	0.67
Demiroglu 2020^40^	German	100 (84)	Depressed: 44 records. Not depressed: 56 records	Interview, general questions (e.g., “What is your favorite dish?”)	Same as above	BDI	SVM	Leave-one-out	0.85*	0.77			0.89	0.75
Gao 2024a^41^	Chinese	156	Depressed: 77 Not depressed:, 79	Responses to four questions about recent events, sleep, mood, and suicidal tendencies	Transformer (BERT and an improved TextCNN)	Medical records	Dual-branch BERT + improved TextCNN model	Train: 94 (60%) Validation: 31 (20%) Test: 31 (20%)	0.942	0.947			0.931	0.964
Guo 2024^42^	Chinese	524	Depressed: 59 Not depressed: 465	Clinical interview	Transformer (EmoLLM + GraphRAG)	HAMD, HAMA	EmoLLM	N/A	0.84	0.49			0.38	0.68
Hayati 2022^43^	Dialectal Malay	53	Depressed: 11 Not depressed: 42	Clinical interview	Transformer (GPT-3)	BDI	GPT3 (They compared its performance using 2–10 examples)	N/A	0.71	0.67				
He 2022 (same data as Yuan 2021)^44^	Chinese	108	Depressed: 54 Not depressed: 54	Picture description and question-answering tasks)	Embedding (Glove)	BDI-II and PHQ-9	GRU based RNN	8:1:1 random train-validation-test split	0.659	0.631			0.688	0.583
Howes 2014^45^	English	882 (167)	Unclear	Interaction with therapist (text-based counseling)	n-gram	PHQ-9	Logistic regression	10-fold cross-validation		0.686				
Iyortsuun 2024^46^ (Same as Shen 2022)	Chinese	162	Depressed: 30 Not depressed: 132	General interview	Transformer (Transformer-based, USE-large)	SDS	BiLSTM + Attention	3-Fold cross-validation	0.606	0.66			0.79	0.58
Joharee 2023^47^	Bahasa Malaysia	511 (172)	Unclear (in the teste set 28 depressed and 23 not depressed)	3 open-ended questions	TF-IDF	BDI-II and PHQ-9	Extra Tree Classifier	Split 70% training and 30% test	0.73	0.63				
Krishnamurti 2022^48^	English	1007 (666)	Not depressed: 48.2% Mild: 38.3% moderate: 10.5% severe: 3.0%	Open-ended questions documenting their pregnancy journey	LIWC; Embedding (Word2Vec); Latent Dirichlet Allocation (LDA), SentiWordNet (SWN)	Edinburgh Postnatal Depression Scale (EPDS)	LASSO regression model	70% training, 15% for prediction (additional 15% were not used)			0.87			
Li 2023^49^	Chinese	387 (329)	Euthymia = 46, mild = 102, moderate = 160, severe = 79	Clinical interview	Transformer (BERT)	HAMD-17	BiLSTM + Self-Attention + Multilayer Perceptron (MLP) + Softmax,	Training = 273 recordings, Test = 114 recordings.	0.86	0.911		0.696	0.921	0.901
Liu 2022^50^	English	219	Depressed: 64 Not Depressed: 155	Text message	LIWC	PHQ-8	Logistic Regression with L2 regularization	leave-one-out			0.72			
Munthuli 2023^51^	Thai	80	Depressed: 40 Healthy control: 40	Clinical interview	Fine-tuned transformer encoder (XLM-RoBERTa)	PHQ-9 and HAM-D	Transformer-based binary classifier (XLM-RoBERTa)	K×L-fold stratified and nested cross-validation	0.9	0.898		0.925	0.921	0.875
Nobles 2018^52^	English	1213 (33)	Suicidality day: 685 Depression Day: 528	Text message	TDF-IDF	Depression: periods where the individual had no suicidal ideation or attempt	DNN	10-fold cross-validation	0.7	0.75		0.56	0.71	0.81
Oh 2024^53^	Korean	166 (77)	Depressed: 60 Other psychiatric illnesses: 17	Clinical interview	Emotional Analysis Module patented by Acryl Inc.	Clinical diagnosis (DSM-5), provided by psychiatrist	XGBoost	Train: 136 Test:30	0.794	0.877	0.85	0.25		0.962
Ohse 2024^54^	German	84	Depressed: 25 Not Depressed: 59	Clinical interview	GPT3.5 fine-tuned	PHQ-8	GPT3.5 fine-tuned	N/A	0.910	0.820			0.850	0.840
Orhan 2019^55^	Turkish	60	Depressed: 30 Healthy control: 30	10-minute free verbal samples of the subjects	Turkish version of the Harvard-III Psychological Dictionary	Structured clinical diagnosis	Bayesian Logistic Regression	Train: 42 (21 for each category) Test: 18 (9 for each set)	0.89					
Parkeaw 2025^56^	Thai	373	Low risk: 261, High risk: 112	SCT consisted of 34 items covering four key depression-related domains: 1) family, 2) society, 3) health, and 4) self-concept	LLM (LLama3.1) was used to extract sentiment scores	PHQ-9	Random forest	5-fold cross-validation	0.786				0.782	
Pérez-Toro 2022^57^	Spanish	60	Depressed Parkinson’s Disease patients (D-PD): 25 Non-depressed Parkinson’s Disease patients (ND-PD): 35	Free response prompt (asked to talk about their daily routines)	Transformer (BERT)	Depression item from the MDS-UPDRS	Gaussian Mixture Model-Universal Background Model	Nested leave one out cross-validation	0.67*	0.7	0.7	0.8		0.56
Podina 2025^58^	Romanian	765	Depressed: 397 Not depressed: 367	Clinical interview (with the aiCARE chatbot)	TF-IDF	PHQ-9	Logistic regression	This is a test set for the algorithm that was built in Boian et al., 2025^33^	0.84	0.85		0.78	0.76	0.93
Qin 2025^15^	English	37	Depressed: 17 Control: 20	3 Phases: 1. small talk, 2. semi-structural interview. 3. Demographic questions	LLM (qCammel-13B-GPTQ)	MINI	LLM (qCammel-13B-GPTQ)	N/A	0.81	0.87	0.88			0.80
Ren 2024^59^	English	1070 (94)	Depressed: 570 Not depressed: 500	Interaction with therapist (message-based online therapy)	LIWC; Transformer (BERT)	PHQ-9	Neural network (classification head, unspecified)	Training: 870 Test set: 200 Each of the 94 participants contribute 3 observations for training and one for the test.	0.60	0.59	0.64			
Resnik 2013^60^	English	124	Depressed: 12 Not depressed: 112	Students were asked to “describe your deepest thoughts and feelings about being in college”.	LIWC; Topic modeling (LDA)	BDI	Logistic Regression	Split to train (94) and test (30)	0.80*	0.50			0.50	0.50
Rutowski 2020^61^	English	15,950 (11,000)	Depressed: 4259 Not depressed: 11,691	Participants interacted with an app that presented questions on different topics, such as “work” or “home”.	Transfer learning, implemented via ULMFiT	PHQ-8	LSTM	Split to train (80%) and test (20%)	0.75		0.82	0.75		0.75
Shen 2022^62^	Chinese	162	Depressed: 30 Not depressed: 132	Interviews	Embedding (ELMo)	PHQ-8 SDS	BiLSTM with Attention	3-fold CV		0.65			0.65	0.66
Shin 2022^63^	Korean	166	Depressed: 83 Healthy control: 83	Clinical interview	LIWC; Bag-of-words	MINI	Naive bayes	80/20 split	0.83		0.91	0.96		0.70
Shin 2024^64^	Korean	428 (91)	Depressed: 73 Not depressed: 357	Daily diary	Transformer Gpt3.5_ft_CoT (fine-tuned models, Chain-of-thought)	PHQ-9 and Beck Scale for Suicide Ideation (BSS)	Gpt3.5_ft_CoT (fine-tuned models, Chain-of-thought)	N/A	0.90	0.69		0.95	0.75	0.64
Smirnova 2018^65^	Russian	201	Depressed: 124 Healthy control: 77	Free response prompt; (Participants wrote narratives on the topic ‚”The current state of life and future expectations)	Lexico-semantic features: metaphors, similes, informal words, repetitions Syntactic features: sentence types, word order, ellipses Lexico-grammatical features: pronouns, verb tenses/forms	Clinical psychiatric interviews coded using ICD-10 diagnostic criteria	Linear discriminant analysis	Mention cross validation, but not clear which type	0.99					
Smirnova 2019^66^	Russian	201	Same as above	Same as above	Component lexis analysis	HDRS-21	Linear discriminant analysis	Mention cross validation, but not clear which type	0.96					
Sood 2023^67^	English	626	Depressed: 152 Not depressed: 474	Clinical interview [Combination of 3 data sets: DAIC, E-DAIC and EATD corpus (originally chines but translated to English)]	TDF-IDF	PHQ-8 and SDS	SVM	Training set: 399 Development set: 108 Test set: 119 (34 depressed)	0.90*	0.82			0.83	0.83
Tao 2023^68^	Chinese	139	Depressed: 64 Anxious: 75	Interaction with chatbot asking about daily activities	Transformer (ChatGPT)	Psychiatrist diagnosis	ChatGPT	N/A	0.68	0.71			0.69	0.72
Tlachac 2020^69^	English	162	Depressed:55 Not Depressed: 107	Text message	Lexical category features via Empath; POS tag frequencies; Sentiment scores (polarity and subjectivity); Volume features: number of messages, words, characters	PHQ-9	Logistic Regression	5-fold cross-validation	0.804*	0.806		0.742	0.728	0.925
Tlachac 2022a^70^	English	302	Depressed: 142 (47.0%) Not depressed: 160 (53.0%)	Free response prompt	Transformer (BERT); Part-of-speech (POS) tagging Lexical category features via Empath	PHQ-9	BERT-LSTM (a variation of BERT incorporating a Long Short-Term Memory layer)	Training set: 218 Test set: 84 (27.8%)	0.55	0.67		0.17	0.51	0.97
Tlachac 2022b^71^	English	3,000 (unclear of how many participants)	Unclear	Text message	Transformer (BERT)	PHQ-9	Fine-tuned BERT classifier	Training: 2400 (1,200 messages per class) Testing: 600 (300 messages per class)	0.711					
Tlachac 2022^72^	English	88	Depressed: 53 Not depressed: 35	Text message	Lexical category; Frequency features; BOW	PHQ-9	Logistic Regression	leave-group-out cross validation	0.71	0.79		0.4		0.93
Weber 2025^73^	German	126 (65 from 44 participants, 61 synthetic)	n/a	Clinical interview	Transformer (BERT-base-German-cased)	MADRS	Linear regression	5-fold cross validation	0.83					
Wright-Berryman 2023^74^	English	2416 (1433)	Depressed: 863 Not depressed:1553	Clinical interview	TF-IDF	PHQ-9	SVM	Leave-one-subject-out cross-validation	0.69		0.77	0.04	0.68	0.55
Xue 2024^75^ (Same as Shen 2022)	Chinese	162	Depressed: 30 Not depressed: 132	EATD-Corpus (general interview)	Transformer (BERT)	SDS	Fine-tuned BERT model with fully connected layers	Does not specify what type of validation was used		0.72			0.66	0.80
Ye 2021^76^	Chinese	160	Depressed: 80 Not depressed: 80	Clinical interview	Embedding (Word2vec)	HAMD	One-hot Transformer	of 5-fold cross validation.	0.882	0.874				
Yuan 2021^77^	Chinese	108	Depressed: 54 Not depressed: 54	Picture descriptions and responses to 30 questions.	Embedding	BDI-II and PHQ-9	Text Recurrent Encoder (TRE)	8:1:1 random train-validation-test split	0.659	0.651			0.688	0.583
Zhang 2024a^78^	Chinese	240	Depressed: 120 Not depressed: 120	Clinical interview	BERT Chinese pre-training model with Multi-Head Attention (MHA) module	PHQ-9	Fully connected deep learning classifier	Training set: 168, Test set: 72	0.64	0.64			0.64	0.64
Zou 2023^79^	Chinese	78	Depressed: 26 Not depressed: 52	Clinical interview	Transformer (Chinese BERT)	MINI	Logistic Regression	5-fold cross-validation	0.92*	0.93	0.99		0.87	0.93
Studies Reporting Continuous Outcomes														
Study	Language	N	Source of text	Features extraction method		Outcome	Type of classification model	Validation	MAE		RMSE		R2	
Morales 2016^80^	German	138 (84)	Interview on everyday life aspects	LIWC; n-gram; Part-of-Speech (POS); Text-based speech rate features		BDI-II	SVM	leave-one-out cross-validation	7.56		9.21		0.526	
Ozkanca 2018^81^	Turkish	70	Open-ended questions (neutral, positive, and negative questions)	Manual sentiment tagging (positive/negative/neutral), number of responses per sentiment, average utterance length, speech rate, features computed separately for positive/negative/neutral questions (15 total features)		BDI-II	SVR	Leave-one-out			10.3			

**Note**: The number in the “N” column represents the total number of text observations, with the value in parentheses indicating the number of participants from whom these observations were collected. The accuracy result marked with a * has been computed by us. BiLSTM: Bidirectional Long Short-Term Memory; LIWC: Linguistic Inquiry and Word Count; CNN: Convolutional Neural Network; TF-IDF: Term Frequency–Inverse Document Frequency; PHQ-9: Patient Health Questionnaire–9; BERT: Bidirectional Encoder Representations from Transformers; IIFDD: Intra- and Inter-modal Fusion Model for Depression Detection; SVM: Support Vector Machine; LASSO: Least absolute shrinkage and selection operator; BDI-II: Beck Depression Inventory–II; GRU: Gated Recurrent Unit; RNN: Recurrent Neural Network; LDA: Latent Dirichlet Allocation; POS: Part-of-speech; HAMD: Hamilton Depression Rating Scale; DNN: Deep Neural Net; XGBoost: Extreme Gradient Boosting; MDS-UPDRS: Movement Disorders Society Unified Parkinson’s Disease Rating Scale; ULMFiT: Universal Language Model Fine-tuning; SDS: Self-rating Depression Scale questionnaire; HDRS-21: Hamilton Depression Rating Scale-21; SVR: Support Vector Regression; SCT: Sentence Completion Test; MADRS: Montgomery-Åsberg Depression Rating Scale.

**Table 2:** Description of Studies That Use DAIC Dataset

First author	Features extraction method	Outcome type	Type of classification model	Accuracy	F1	Precision	Recall/Sensitivity	MAE	RMSE
Agarwal 2022^82^	Embedding (GloVe)	Binary	MV-IA-Mean	0.72	0.73	0.74	UAR: 0.72		
Agarwal 2024^83^	Embedding (Sentence embeddings from all-mpnet-base-v2; graph built using cosine similarity between embeddings)	Binary	GCN + Transformer multi-head attention	0.83	0.81	0.80	UAR: 0.82		
Al-Hanai 2018^84^	Embedding (Word2Vec)	Binary	LSTM		0.67	0.57	0.8	5.18	6.38
Ansari 2023^85^	Count vectorization	Binary	LR and LSTM	LR: 0.748, LSTM: 0.73	LR: 0.67, LSTM: 0.61				
Burdisso 2023*^86^	TF-IDF; PMI (Pointwise Mutual Information).; PageRank	Binary	node-weighted GCN		0.84				
Cao 2022^87^	Transformer (BERT)	Binary	BERT	0.91					
Chen 2024^88^	TF-IDF; PMI (Pointwise Mutual Information).; PageRank	Binary	GCN		0.84				
Correia 2016^89^	Embedding (GloVe)	Binary	SVM	Per sentence: 0.533 Per interview: 1.00					
Dang 2017^90^	SALAT; siNLP; TAALES; SEANCE; ANEW; EmoLex; SenticNet; Lasswell	Cont.	SVR					4.98	6.02
Danner 2023^91^	Transformer (BERT)	Binary	BERT		0.82	0.83	0.82		
Fang 2023^92^	Transformer (USE)	Cont.	Bi-LSTM with an attention mechanism					3.61	4.76
Firoz 2023a^93^	BoW TF-IDF Embedding (Word2Ve, FastTex)	Binary	Ensemble model of CNN-LSTM-and Bi-LSTM	0.80					
Firoz 2023b^94^	Transformer (BERT); Counts of absolutist language (e.g., always, never, completely)	Cont.	LSTM					5.65	9.45
Flores 2023^24^	Transformer (BERT)	Binary	LSTM		0.72				
Guo 2024^95^	Transformer (BERT)	Binary	PTDD	0.69	0.60	0.48	0.73		
Hadzic 2024^96^	GPT4	Binary	GPT4		0.71	0.81	0.70		
Hong 2022^97^	Embedding (GRL using Schema Encoders)	Cont.	Schema-Based Graph Neural Network					3.76	
Iyortsuun 2024^45^	Transformer (Transformer-based, USE-large)	Binary and cont.	BiLSTM + Attention	0.727	0.78	0.80	0.76	3.96	
Jo 2022*^98^	Embedding (unclear the exact type)	Binary	CNN	0.8171	0.8101	0.80	0.8205		
Kokkera 2023^99^	Word frequencies POS tags Sentiment scores	Binary	RF	0.40	0.40	0.44	0.43		
Lam 2019^100^	Manual topic modelling + augmentation + embedding + Transformer	Binary	Transformer architecture		0.78	0.91	0.83		
Lau, 2021^101^	Transformer (BERT)	Binary and cont.	BiLSTM + attention		0.83	0.83	0.83	4.23	5.32
Lau 2023^102^	Transformer (BERT and RoBERTa)	Cont.	BiLSTM + attention					4.17	0.02
Li 2022a^103^	Embedding (from scratch)	Binary	biLSTM + RNN network	0.745	0.706	0.701	0.715		
Li 2022b^104^	Transformer (BERT) (utterance-based)	Binary	BiLSTM + attention with an MLP-Softmax classifier		0.78		UAR: 0.79		
Li 2023^105^	Part-of-Speech (POS); Named Entity Recognition (NER); Embedding (GloVe)	Binary	BiLSTM		0.79	0.69	0.80		
Lin 2020^106^	Embedding (Elmo)	Binary	BiLSTM + Attention		0.83	0.83	0.83		
Lopez-Otero 2017^107^	Embedding (GloVe)	Binary	SVM	0.857	0.730				
Lorenc 2022^108^	Embedding and transformer (USE5, DAN, sBERT)	Binary	Chunk-based biLSTM model				UAR: 0.803		
Lu 2023^109^	Transformer (BERT)	Binary	BERT		0.76				
Rodrigues Makiuchi 2019^110^*	Transformer (BERT)	Cont.	8 CNN blocks-LSTM					4.22	
Mallol-Ragolta 2019^111^	Embedding (GloVe)	Binary	HCAN		0.63		UAR: 0.66		
Mao 2023^112^	Embedding (GloVe)	5-levels classification	BiLSTM	0.968	0.971				
Milintsevich 2023^113^	Transformer (RoBERTa)	Binary, 5-levels classification and cont.	BiLSTM + Attention		**Binary:** Micro-F1 = 0.766 Macro-F1 = 0.739 **5-Class:** Micro-F1 = 0.426 Macro-F1 = 0.270			3.78	
Niu 2021^114^	Embedding (GloVe)	Binary and cont.	Hierarchical context-aware graph attention model	0.77		0.70	0.82	3.73	4.8
Pampouchidou 2016^115^	LIWC; Total number of words and sentences Average sentence length Laughter-to-word ratio Depression-related word ratio: ANEW Mean and SD of pleasure, arousal, dominance ratings Word frequency	Binary	Decision Tree		Depressed: 0.23 Not depressed: 0.79			8.99	10.75
Prabhu 2022^116^	Embedding (Word2vec pretrained)	Binary	LSTM	0.823					
Qureshi 2019^117^	Embedding (from scratch, feature learning via an LSTM encoder)	Continuous and 5-class	DNN	0.67	0.53			3.90	4.96
Qureshi 2020^118^	Transformer (USE)	Cont. and 5-level class	LSTM	0.667	0.62			Class: 0.66 Cont: 3.81	Class: 1.23 Cont: 4.70
Qureshi 2021^119^	Transformer (USE)	Cont.	LSTM					3.78	4.88
Rasipuram 2022^120^	Transformer (GPT2)	Cont.	BiLSTM					3.21	4.25
Ray 2019^121^*	Transformer (USE)	Cont.	stacked BiLSTM + feedforward network					4.02	4.73
Rinaldi 2020^122^	Embedding (GloVe)	Binary	Joint Latent Prompt Categorization (JLPC)		0.604				
Rohanian 2019^123^	Embedding (GloVe)	Binary and cont.	LSTM		0.69	0.68		4.98	6.05
Sadeghi 2023^124^*	Transformer (GPT-3.5-Turbo and DepRoBERTa)	Cont.	SVR with a polynomial (poly) kernel					4.26	5.36
Sadeghi 2024^125^*	Transformer (GPT-3.5-Turbo (prompt asking the model to describe the interview + DepRoBERTa and GPT-3.5-Turbo response to 11 questions on the interview)	Cont.	SVR					3.86	4.66
Samareh 2018^126^	Basic linguistic stats (e.g., word count); Dictionary based depression-related word ratio; Sentiment features (AFINN)	Cont.	RF regression with confidence-based decision-level fusion.					4.78	5.59
Senn 2022^127^	Transformer (BERT and RoBERTa)	Binary	Ensemble of BERT, RoBERTa, DistilBERT		0.62		0.64		
Shen 2022^61^ (used also eatd corpus)	Embedding (ELMo)	Binary	BiLSTM with Attention		0.83	0.83	0.83		
Stasak 2017^128^	Word Affect Features: single affect word-rating reference, such as the General Index	Binary	decision tree classification	0.82					
Stepanov 2018^129^	BOW	Cont.	SVR					4.88	5.83
Sun 2017^130^	Selected key phrases related to symptoms	Cont.	RF		0.55	0.40	0.89	3.87	4.98
Tlachac 2022^70^	Transformer (BERT)	Binary	fine-tuned BERT classifier	0.48					
Toto 2021^131^	Transformer (BERT)	Binary	LSTM		0.67				
Marriwala 2023^132^	Embedding (Word2vec)	Binary	CNN	0.8	0.6	0.63	0.68		
Van Steijn 2022^133^*	LIWC; Transformer (BERT); Sentiment; speech rate; Repetition rate; Con dence score	Cont.	KELM						6.06
Villatoro-Tello 2021^134^*	Lexical Availability	Binary	MLP (Train the model on E-DAIC and tested on DAIC-WOZ)		0.83	0.87	0.81		
Williamson 2016^135^	Embedding (GloVe); Topics	Binary and cont.	SVR		0.84			3.34	4.46
Xezonaki 2020^136^	LIWC; TDF-IDF; Embedding (GloVe); Affective lexica (AFINN, Bing Liu, MPQA, Emolex, SemEval15)	Binary	Hierarchical Attention Network with Lexicon and Summary Integration		0.70	0.70			
Xia 2024^137^	Embedding (Word2vec)	Binary	BiLSTM-GNN	0.64	0.60	0.585	0.584		
Xiao 2021^138^	Transformer (BERT)	Binary	BERT				0.70		
Xu 2023^139^*	Transformer (BERT)	Binary	Two-layer STM network		0.82	0.81	0.83		
Xue 2024^73^	Transformer (BERT)	Binary	Fine-tuned BERT model with fully connected (FC) layers		0.85	0.79	0.92		
Yadav 2023^140^	Embedding (Word2Vec, ELMo); Transformer (BERT)	5-levels class	BGRU model with two Fully Coupled (FC) networks as output layers		0.923	0.929	0.928		
Yang 2017a^141^	Embedding (PV); Global structural and behavioral text features (e.g., Number of words)	Binary	SVM		Depressed: 0.667 Not depressed: 0.885	Depressed: 1.000 Not depressed: 0.793	Depressed: 0.50 Not depressed: 1.00		
Yang 2017b^142^	Embedding (PV)	Cont.	DCNN and DNN					Female: 3.750 Male: 3.525	Female: 4.361 Male: 4.406
Yang 2018^143^	Embedding (PV)	Binary	SVM	0.75					
Yang 2019^144^	Embedding (Doc2vec) and Text Convolutional Neural Network	Binary	SVM	0.72					
Zhang 2020a^145^*	Embedding (PV or doc2vec)	Binary and cont.	Multitask Deep Neural Network (DNN)	0.839	0.907				4.66
Zhang 2020b^146^	Transformer (BERT); Key phrase matching	Binary	bidirectional variable-length LSTM model		0.81	0.82	0.8		
Zhang 2024b^147^	Transformer [Sentence-BERT (nli-bert-large)]	Binary	BiLSTM		0.87				
Zhang 2024c^148^	Transformer (T5-Encoder and BERT)	Binary, 3-levels, 5-levels	T5 + BERT dual-branch fusion	Binary: 0.8913 3-level: 0.6739 5-level: 0.5435	Binary: 0.8276 3-level: 0.6677 5-level: 0.5259	Binary: 0.80	Binary: 0.857	5.283	
Zhao 2022^149^	n-gram	Cont.	Transformer-based architecture with self-attention and feed-forward layers					5.03	5.95

**Note**:

* indicate studies using the E-DAIC dataset; all others are based on DAIC-WOZ. Since only two studies reported AUC and three studies reported specificity, these metrics were removed from the table. MV-IA-Mean: Multi-view model with inter-view attention coupled with the mean function; GCN: Graph Convolutional Network; *SALAT*: Suite of Linguistic Analysis Tools: This open-source toolkit was used to extract various linguistic and word affect features from transcripts. *siNLP*: Simple Natural Language Processing Tool; *TAALES*: Tool for Automatic Analysis of Lexical Sophistication; *SÉANCE*: Sentiment Analysis and Cognition Engine; ANEW: Affective Norms for English Words; *EmoLex*: This provided features based on token words related to eight emotion types (e.g., anger, anticipation, disgust, fear, joy, sadness, surprise, trust); *SenticNet*: This provided features based on nearly 13,000 token words, evaluating perceptual polarity norms for aptitude, attention, pleasantness, and sensitivity; *Lasswell*: This provided 146 features from 63 different word lists categorized by eight semantic characterizations, with a particular interest in the well-being category; *BERT*: Bidirectional Encoder Representations from Transformers; SVR: Linear Support Vector Regression; *Bi-LSTM*: Bi-directional LSTM; SVM: Support Vector Machine; NB: Naïve Bayes; LR: Logistic Regression; LSTM: Long Short Term Memory; USE: Universal Sentence Encoder; *BoW*: Bag of Words; TF-IDF: Term Frequency-Inverse Document Frequency; PTDD: Prompt-based Topic-modeling method for Depression Detection; RF: Random Forest; UAR: Unweighted Average Recall; USE5: USE Transformer-based; DAN: Deep Averaging Network a simpler sentence embedding model; *sBERT*: Sentence-BERT Transformer fine-tuned for sentence similarity; HCAN: Hierarchical Contextual Attention Network; POS: Part-of-speech; DepRoBERTa: fine-tuned RoBERTa language model, which is specifically designed for depression detection; CNN: Convolutional Neural Network; KELM: Kernel Extreme Learning Machine; BGRU: Bidirectional Gated Recurrent Unit; DCNN :Deep Convolutional Neural Network; DNN: Deep Neural Network; MLP: Multi-layer Perceptron; MDSD-T5: the T5-based (Google encoder–decoder Transformer) branch of the MDSD-FGPL system; PV: paragraph vector, an extension of Word2Vec.

## Data Availability

Data and materials underlying the findings of this study are publicly available at [https://osf.io/x7tm9/overview](https:/osf.io/x7tm9/overview).

## Footnotes

1. Because the DAIC dataset and its derivatives were repeatedly used across numerous studies, including overlapping samples and similar recording procedures, their inclusion could bias descriptive summaries toward the characteristics of that single corpus. Therefore, for dataset characteristics section, DAIC-based studies are summarized separately from those using unique datasets to better represent the diversity of data sources included in the review.

2. Many studies tested multiple NLP feature extraction and classification methods. The counts reported here represent the best-performing approach in each study rather than the only method used. For example, complex models were compared with simpler or traditional approaches to test improved performance.
